# A novel substrate for arrhythmias in Chagas disease

**DOI:** 10.1371/journal.pntd.0009421

**Published:** 2021-06-02

**Authors:** Artur Santos-Miranda, Julliane V. Joviano-Santos, Jaqueline O. Sarmento, Alexandre D. Costa, Allysson T. C. Soares, Fabiana S. Machado, Jader S. Cruz, Danilo Roman-Campos

**Affiliations:** 1 Department of Biophysics, Universidade Federal de São Paulo, São Paulo, Brazil; 2 Department of Pharmacology and Physiology, Biological Sciences Institute, Universidade Federal de Minas Gerais, Minas Gerais, Brazil; 3 Department of Biochemistry and Immunology, Biological Sciences Institute, Universidade Federal de Minas Gerais, Minas Gerais, Brazil; University of Georgia, UNITED STATES

## Abstract

**Background:**

Chagas disease (CD) is a neglected disease that induces heart failure and arrhythmias in approximately 30% of patients during the chronic phase of the disease. Despite major efforts to understand the cellular pathophysiology of CD there are still relevant open questions to be addressed. In the present investigation we aimed to evaluate the contribution of the Na^+^/Ca^2+^ exchanger (NCX) in the electrical remodeling of isolated cardiomyocytes from an experimental murine model of chronic CD.

**Methodology/Principal findings:**

Male C57BL/6 mice were infected with Colombian strain of *Trypanosoma cruzi*. Experiments were conducted in isolated left ventricular cardiomyocytes from mice 180–200 days post-infection and with age-matched controls. Whole-cell patch-clamp technique was used to measure cellular excitability and Real-time PCR for parasite detection. In current-clamp experiments, we found that action potential (AP) repolarization was prolonged in cardiomyocytes from chagasic mice paced at 0.2 and 1 Hz. After-depolarizations, both subthreshold and with spontaneous APs events, were more evident in the chronic phase of experimental CD. In voltage-clamp experiments, pause-induced spontaneous activity with the presence of diastolic transient inward current was enhanced in chagasic cardiomyocytes. AP waveform disturbances and diastolic transient inward current were largely attenuated in chagasic cardiomyocytes exposed to Ni^2+^ or SEA0400.

**Conclusions/Significance:**

The present study is the first to describe NCX as a cellular arrhythmogenic substrate in chagasic cardiomyocytes. Our data suggest that NCX could be relevant to further understanding of arrhythmogenesis in the chronic phase of experimental CD and blocking NCX may be a new therapeutic strategy to treat arrhythmias in this condition.

## Author summary

Chagas disease (CD), caused by the parasite *Trypanosoma cruzi*, is a neglected disease that induces heart failure and arrhythmias in approximately 30% of patients during the chronic phase of the disease. There are several substrates for arrhythmias in the heart. Some of them involve changes in the electrical properties of cardiomyocytes, the working cells of the heart. In our study we evaluate the potential involvement of Na+/Ca2+ exchanger (NCX) in the arrhythmic phenotype of cardiomyocytes isolated from mice infected with *Trypanosoma cruzi*, between 180- and 200- days post-infection, which is considered the chronic phase of CD in this animal model. In our study we found several arrhythmogenic membrane potential oscillations during action potential measurements, in rest and using a protocol to simulate a pause after a tachycardia. Using pharmacological approach, we determine that NCX significantly contributed to the arrhythmogenic phenomena observed. Thus, in our study we demonstrate that NCX may be relevant to the cellular arrhythmogenic profile observed in cardiomyocytes during the chronic phase of experimental CD and blocking NCX may be a new therapeutic strategy to treat arrhythmias in this condition.

## Introduction

Chagas disease (CD) is a vector-borne disease caused by the parasite *Trypanosoma cruzi* that affects 6 to 7 million people worldwide, mostly in Latin America. Although the original route of transmission occurs through the triatomine vector, additional routes, including contaminated food and vertical transmission further contributes to increase the spread of the disease [1]. In 2016 it was estimated that in the United States there were approximately 238.000 reported cases of CD [2], but the number may be higher [3]. Around 30% of all infected individuals experience severe cardiac complications during the chronic phase of the disease leading to Chagasic CardioMyopathy (CCM). Clinical manifestations include non-sustained and sustained ventricular tachycardia and heart failure that, if not properly dealt with, will culminate in death [4]. Despite the severity of CCM, it is rather difficult to indicate proper therapeutic agents due to the incomplete knowledge of cellular biophysical mechanisms responsible for the generation of cardiac arrhythmias.

In experimental models of CD it was found that cardiomyocytes from infected mice displayed severe electromechanical remodeling [5–8]. Major changes were observed in action potential (AP) repolarization and a consistent reduction of L-type Ca^2+^ current density [6,7,9,10]. However, there are still important and relevant questions to be addressed in the context of the molecular pathophysiology of CCM. Thus, in the present study, an experimental murine model of chronic CCM was used to directly access the possible contribution of Na^+^/Ca^2+^ exchange current (I_NCX_) in the electrical remodeling of cardiomyocytes during chronic experimental CD [5,7].

## Methods

### Ethics statement

All animal related procedures were previously approved by the Institutional Animal Care and Use Committee (protocol #1948230414). All animal experiments were in accordance with the ARRIVE guidelines and were carried out in accordance with the U.K. Animals (Scientific Procedures) Act, 1986 and associated guidelines, EU Directive 2010/63/EU for animal experiments.

### Animals

We used male C57BL/6 mice 8 weeks-old obtained from CEBIO (ICB, UFMG, Belo Horizonte, MG, Brazil). Control and experimental groups were studied between 180- and 200- days after saline injection or post infection (d.p.i.).

### Infection

The Colombian strain of *T*. *cruzi* (DTU TcI) [11] was used in all experiments. Trypomastigotes were maintained by blood passage in Swiss mice every 7 days. Trypomastigotes were obtained from heparinized blood, counted, and used for infection. Mice were injected in the peritoneal cavity with 100 trypomastigotes, as previously described. Control mice received the same treatment, except by the absence of *T*. *cruzi*. [6,7,10,12].

### Cardiomyocyte isolation

Freshly isolated left ventricular cardiomyocytes (LVC) were obtained following a previously described method [13]. After isolation, cardiomyocytes were kept in Tyrode’s solution at room temperature (25°C). Experiments were conducted up to 4 h after LVC isolation. Usually 60–80% of viable LVC was obtained after cell isolation.

### Cellular electrophysiology

Whole-cell patch-clamp recordings were obtained using an EPC-10 patch-clamp amplifier (HEKA, Holliston, Massachusetts, USA) in the voltage- and current-clamp modes [6]. Glass pipettes were pulled with 0.5–1.5 MΩ tip resistance and cells with series resistance higher than 8 MΩ were not considered in the analysis. To achieve better voltage control, all ion current measurements were electronically compensated for series resistance (60–70%). In all records, cells were bathed with regular Tyrode’s solution. After break-in, cells were kept resting for 2–3 minutes, in order to allow proper equilibration. For internal and external solutions see S1 Table.

### Action potential and pause-induced transient current recordings

Action potentials (AP) were triggered using a rectangular (5–7 ms duration) depolarizing current pulses (1 nA). Electrical stimulation frequencies were set at 0.2 and 1 Hz. AP recordings were sampled at 10 kHz. Using Clampfit (Molecular Devices, v10.5) we analyzed time to 50 and 90% of AP repolarization (APR_50_ and APR_90_, respectively), maximal rate of AP depolarization (V/s), overshoot (mV) and resting membrane potential (mV). Stimulation protocols designed to mimic mouse ventricular AP were used to explore whether membrane currents underlying spontaneous activity could be detected under the same experimental conditions used during conventional current-clamp experiments.

Transient inward current (I_Ti_) area was calculated by defining a baseline diastolic current and measuring the area of 9 s of diastolic recording after application of the tachycardia protocol. For some experiments, AP and I_Ti_ were recorded prior and after superfusion of extracellular solution containing Ni+ or SEA0400. Details are given in figure legends.

### Na^+^/Ca^2+^ exchange current recordings, and Sarcoplasmic reticulum Ca^2+^ content

Sarcoplasmic reticulum (SR) Ca^2+^ content was measured in cardiomyocytes held at -80 mV and the membrane potential was depolarized to 0 mV (100 ms) and clamped back to -80 mV. This protocol was repeated every other second for 30 s to achieve steady-state conditions. SR Ca^2+^ content was then estimated by rapidly switching to a solution containing 10 mM of caffeine to cause SR Ca^2+^ release. In the continued presence of caffeine, the SR is unable to reaccumulate Ca^2+^ and extrusion of Ca^2+^ is mainly due to I_NCX_. To directly measure I_NCX_ a ramp protocol (0.012 V/s) from +40 to -70 mV every 10 s was applied. Holding membrane potential was set at -30 mV.

### Real-time PCR for parasite detection in isolated left ventricular cardiomyocytes

Isolated LVC were removed and total RNA was isolated and estimated by real time PCR (polymerase chain reaction). RNA was extracted using TRIzol (Thermo Fisher) and reverse transcription was performed using 500ng of total RNA with iScript Reverse Transcriptase (Biorad) and iScript Reaction Mix (Biorad) in a final reaction volume of 10 μl. Real-time quantitative PCR (qPCR) was performed on an CFX96 real time system (Biorad, Laboratories) using SYBR green PCR master mix (Applied Biosystems) with primers for T. cruzi 18S (18S forward TTGTTTGGTTGATTCCGTCA; 18S reverse CCCAGAACATTGAGGAGCAT) and 18S endogenous (mouse) were (18S forward: CTCAACACGGGAAACCTCA; 18S reverse: CGTTCCACCAACTAAGAACG). The threshold cycle (Ct) and the normalized relative expression levels for the *T*. *cruzi* 18S to mice endogenous 18S (LVC) were determined by the ΔCt method.

### Statistical analysis

Data are presented as means ± standard error of mean (SE), unless when indicated. Statistical significance was determined by paired sample t-test, two-sample t-test, one-way and two-way ANOVA (followed by Tukey’s post-hoc test), after verification of normality using Kolmogorov–Smirnov test. The frequency of “aberrant” AP waveforms and I_Ti_ were tested with Fisher´s exact test. The statistical test is indicated in figure legend. Significance was set at p < 0.05. Data were analyzed using Excel (Microsoft Co. USA) and Origin 8.0 (OriginLab Co. USA).

## Results

### Prolongation of action potential duration is frequency-dependent in experimental Chagas disease

**Fig 1:**
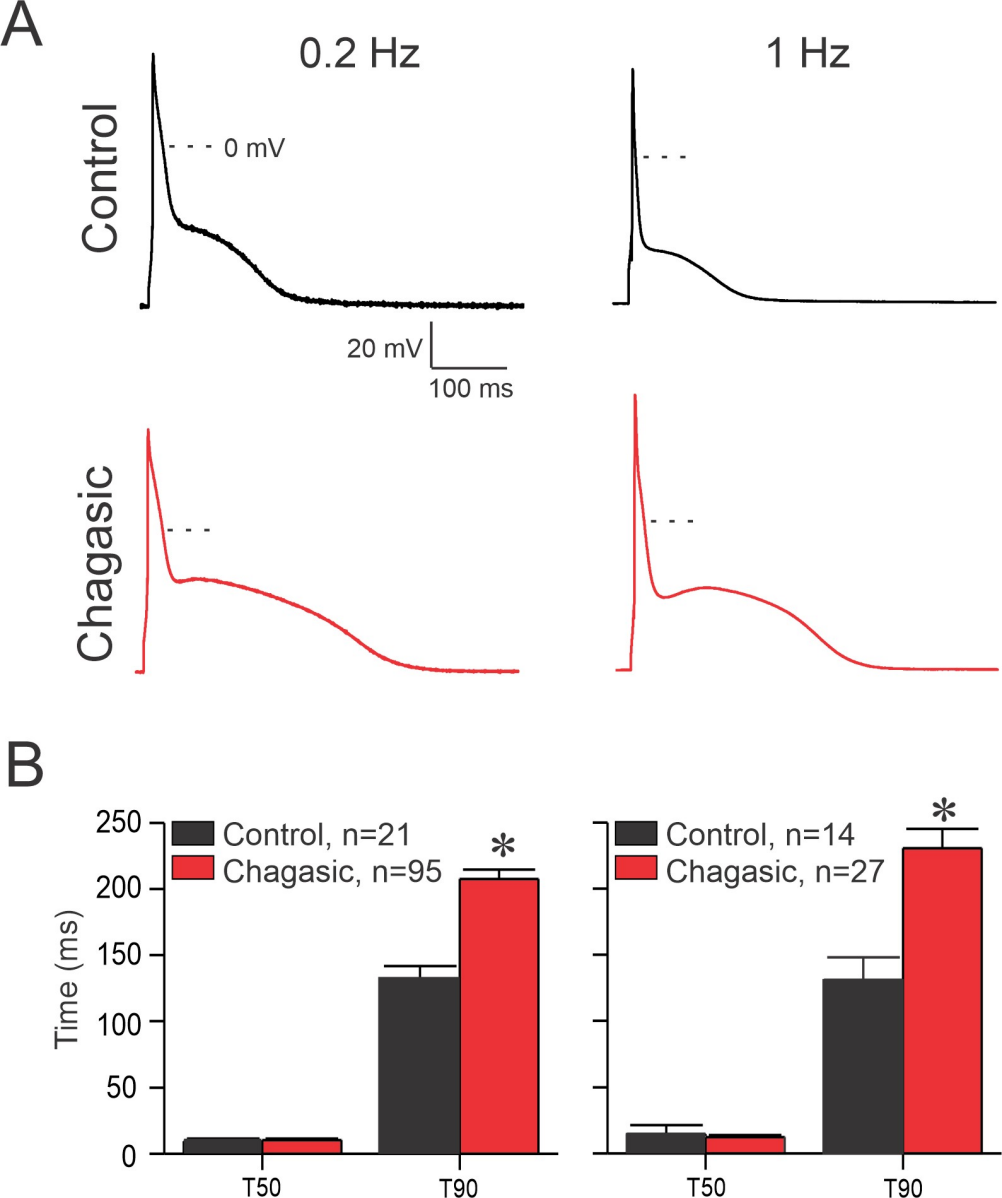
Prolonged action potential (AP) duration in chagasic cardiomyocytes. (A) Representative AP recorded from control (black traces) and infected (red traces) isolated cardiomyocytes paced at 0.2 Hz and 1 Hz. (B) Time required to 50% (T_50_) and 90% (T_90_) of full AP repolarization at 0.2 Hz and 1 Hz. Data were analyzed using Two-way ANOVA. n represents the number of cells. *p<0.05.

Previous studies on experimental CD have found prolongation of cardiomyocyte AP repolarization [6,7,9,10]. However, in most of these studies sarcoplasmic Ca^2+^ was strongly chelated which probably had attenuated the participation of membrane Ca^2+^-dependent conductance. Thus, we decided to revisit the AP waveform in isolated LVC in the chronic phase of experimental CD. Fig 1A shows representative traces of AP recorded at 0.2 and 1 Hz. Fig 1B represents time to 50 and 90% of AP repolarization (APR). At 0.2 and 1 Hz a substantial increase in 90% of APR was observed when comparing healthy and diseased LVC, in agreement with previous results [7,10]. In addition, resting membrane potential was affected by CCM (S2 Table).

### Isolated left ventricular cardiomyocytes from chagasic mice are more susceptible to spontaneous electrical activity

**Fig 2:**
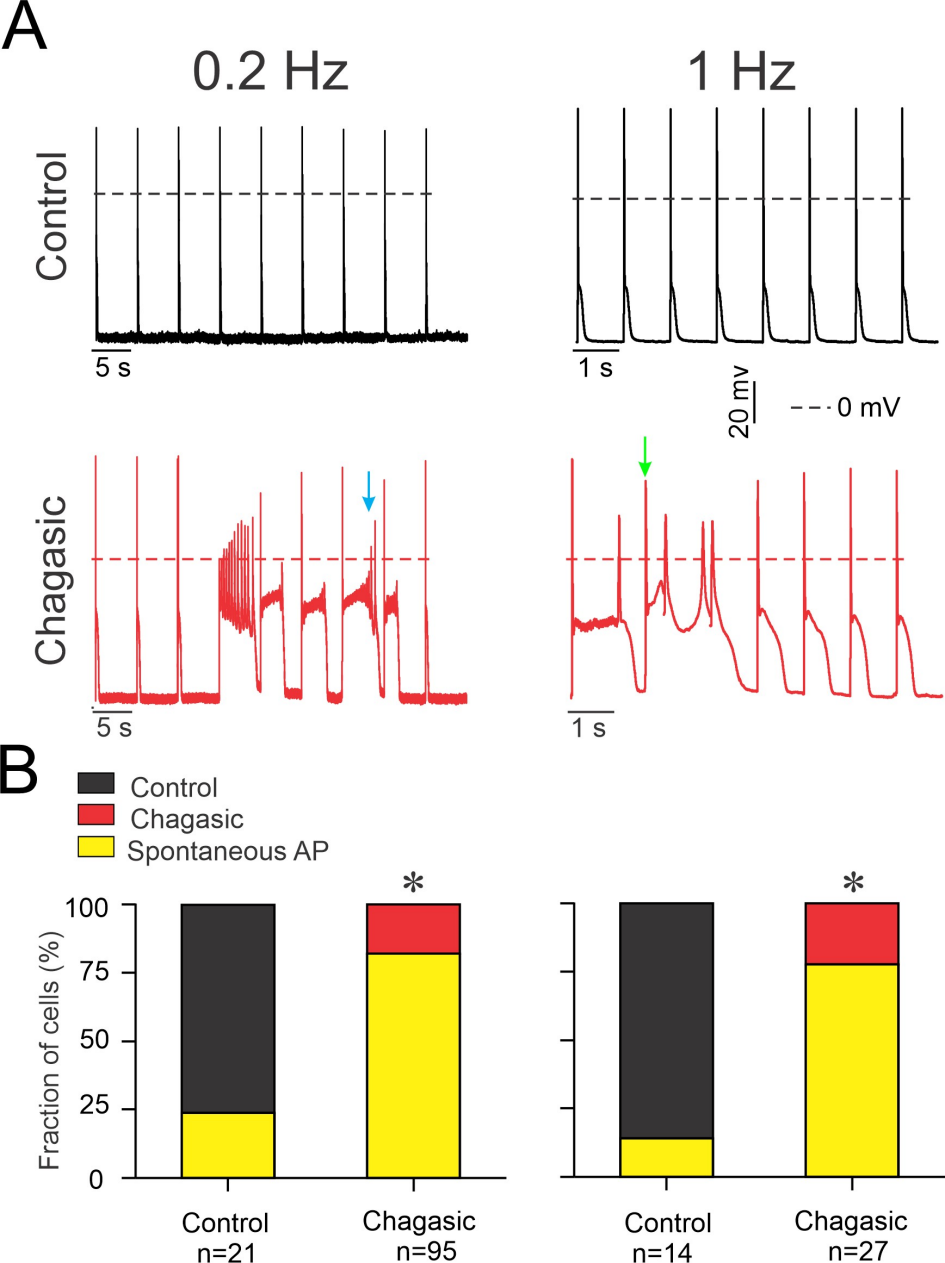
Membrane potential instability due to after-depolarization events in the chronic phase of experimental Chagas disease. (A) Eight to nine consecutive recorded action potentials from control and infected isolated cardiomyocytes paced at 0.2 Hz and 1 Hz. (B) The fraction of cells displaying membrane potential instability triggered by after-depolarizations, either during membrane repolarization (exemplified by the blue arrow) or after full membrane repolarization (exemplified by the green arrow). Data were compared using Fisher’s exact test. n represents the number of cells. *p<0.05.

Fig 2A shows representative 8–9 consecutive APs from healthy and diseased LVCs paced at 0.2 and 1 Hz (top and bottom respectively). In Fig 2 AP instability (i.e early and delayed afterdepolarizations, both subthreshold or able to trigger spontaneous AP) is evident from chagasic LVC, as exemplified by colored arrows. Fig 2B shows the proportion of cells that presented AP instability. Only a minority (between 18% and 22% for 0.2 Hz and 1 Hz, respectively) of LVCs from infected mice showed consistent AP morphology, while for control group the majority of cells (between 76% and 86% for 0.2 Hz and 1 Hz) showed regular AP morphology. In conclusion, LVC from chagasic mice are more susceptible to develop AP instabilities despite pacing frequency (p <0.05).

### Ni^2+^ and SEA0400 restore action potential properties in experimental Chagas disease

**Fig 3:**
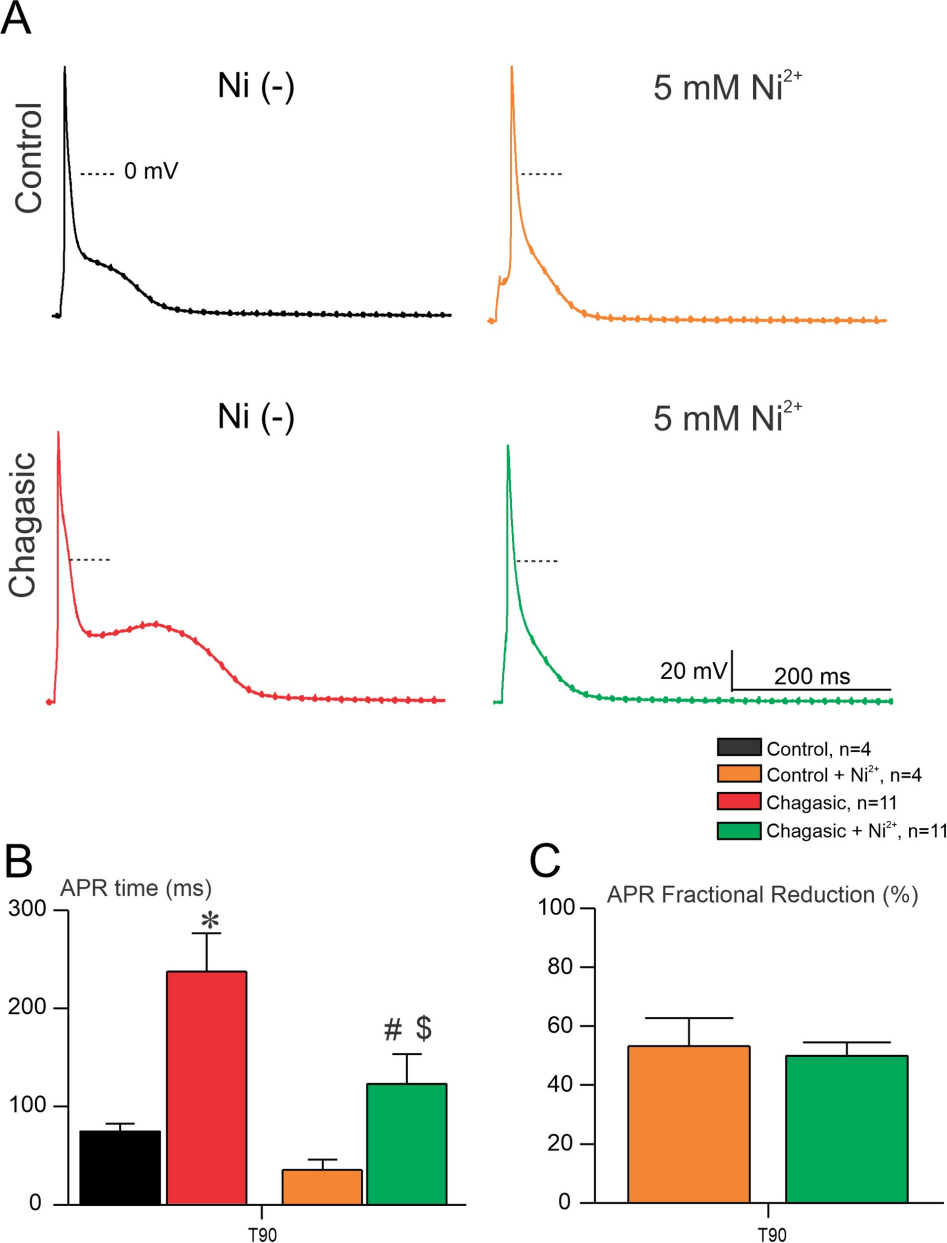
Ni^2+^ shortens action potential duration in chronic phase of experimental Chagas disease. (A) Representative AP recorded from isolated cardiomyocytes from control and infected mice, before and after perfusion of Ni^2+^ (5 mM). (B) Time required to reach 90% (T_90_) of full AP repolarization before and after exposure to Ni^2+^ (5 mM). (C) AP repolarization (APR) fractional reduction taken at 90% after challenge control and chagasic cardiomyocytes with Ni^2+^ at 5mM. Data were compared using one-way ANOVA (B) and Student’s t test (C). n represents the number of cells. * comparing Chagasic to control prior Ni^2+^, # comparing Chagasic prior and after Ni^2+^, $ comparing Chagasic to control after Ni^2+^ (p<0.05).

**Fig 4:**
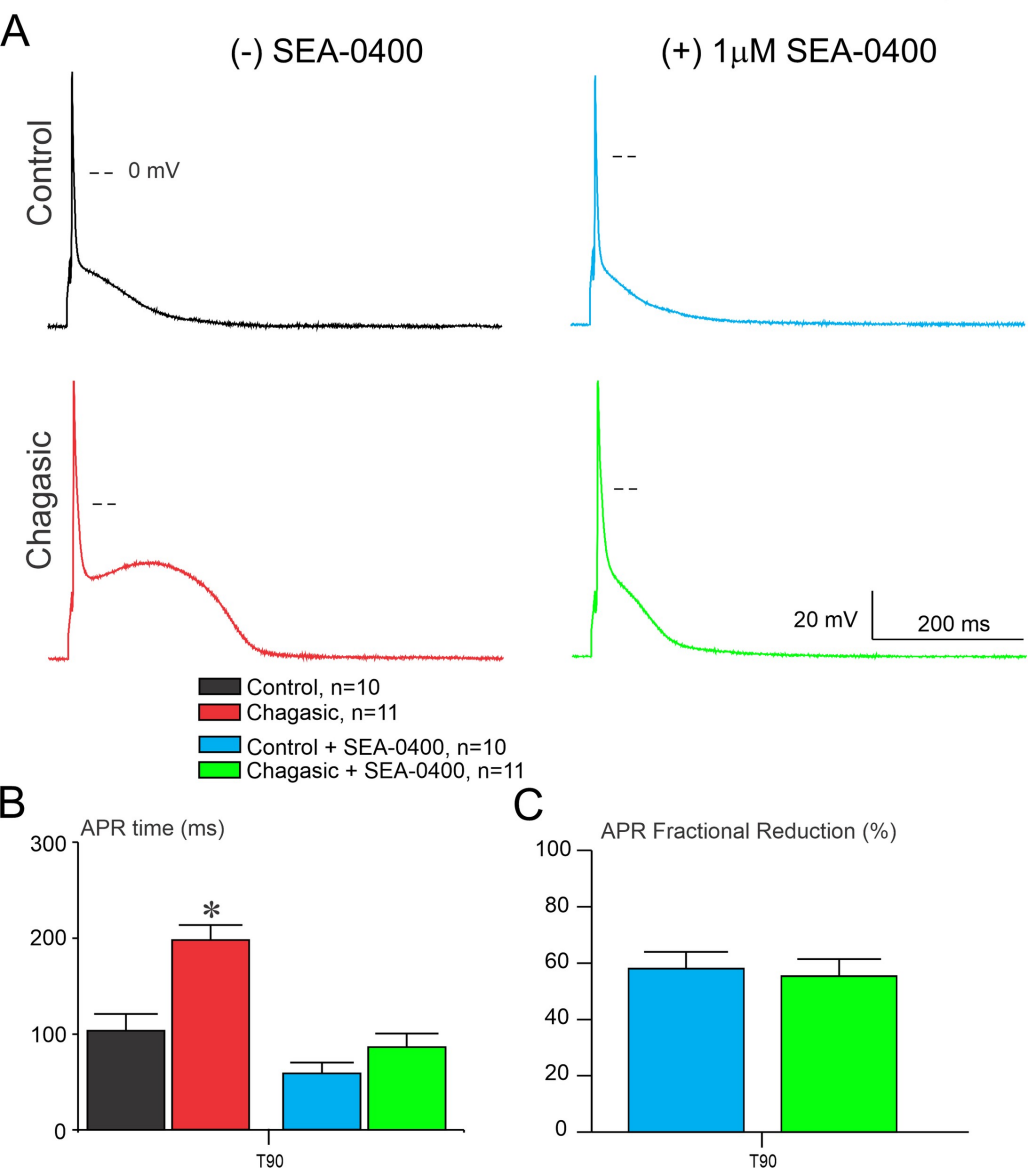
SEA0400 shorts action potential waveform in the chronic phase of experimental Chagas disease. (A) Representative AP recorded from cardiomyocytes isolated from control and infected mice, before and after perfusion of SEA0400 (1 μM). (B) Time required to 90% (T_90_) of full AP repolarization before and after exposure to SEA0400 (1 μM). (C) AP repolarization (APR) fractional reduction taken at 90% after challenge cells with SEA0400 (1 μM). Data were compared using one-way ANOVA (B) and Student’s t test (C). n represents the number of cells. * comparing Chagasic without SEA0400 to all other groups (p<0.05).

There is a myriad of ionic mechanisms that could trigger AP instability, including I_Na,L_ [14, 15], T-type Ca^2+^ current [16], and I_NCX_ [17]. In order to evaluate the possible involvement of I_NCX_ in the electrical remodeling observed, we challenge cells prior and after exposure to Ni^+2^ (non-selective inhibitor of I_NCX_) and we monitored AP waveform. As depicted in Fig 3A, AP paced at 0.2 Hz from healthy LVCs are sensitive to 5 mM Ni^2+^exposure. Importantly, when LVC from chagasic mice were exposed to 5 mM Ni^2+^ we observed a reduction of APR time taken at 90% repolarization (T_90_), as shown in Fig 3B. The mean fractional reduction of T_90_ of control and chagasic mice before and after exposure to Ni^2+^ is displayed in Fig 3C. These AP instabilities were reduced when perfusing Ni^2+^ on LVCs from chagasic mice [6,7,9,10]. Since Ni^2+^ is a non-selective inhibitor of I_NCX_ we performed the same experiment, however, challenging cells with SEA0400, which is considered a selective inhibitor of I_NCX_ [18]. As shown in Fig 4A, healthy and diseased LVC are sensitive to SEA0400. Interestingly, as quantified in Fig 4B, AP waveform from chagasic LVC was strongly affected by SEA0400, as measured by APR_90%_. After exposure of diseased LVC to SEA0400, AP duration was similar to that observed in the control group, which supports the hypothesis that I_NCX_ plays an important role in AP remodeling in CCM. Finally, the mean fractional reduction of T_90_ of control and chagasic mice before and after exposure to SEA0400 is displayed in Fig 4C.

### Pause-induced transient inward current (I_Ti_) is augmented in chagasic cardiomyocytes and is sensitive to Ni^2+^ and SEA0400

**Fig 5:**
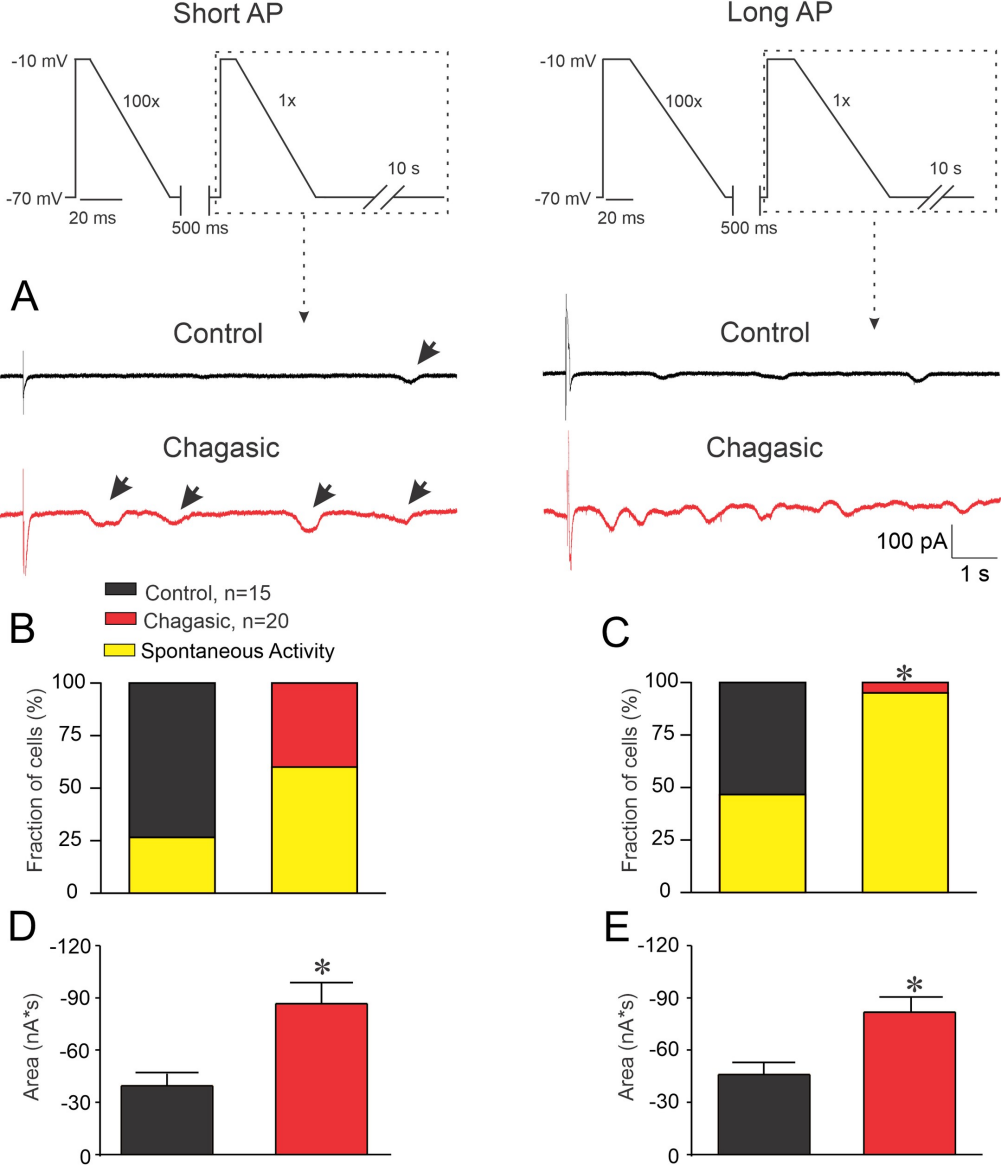
Enhanced susceptibility for the appearance of transient inward current (I_Ti_) in the chronic phase of experimental Chagas disease. The stimulation protocol is depicted at the top of the figure. (A) Current traces following 100 pre-conditioning pulses and a single 500 ms pause using a short pulse (left) and a long pulse (right). Black arrows indicate I_Ti_. (B) and (C) are the percentage of cells showing any identifiable I_Ti_, using short and long pulses, respectively. (D) and (E) are bar graphs summarizing, only for those cells that developed I_Ti_, the calculated integral of the I_Ti_ responses recorded with short and long pulses, respectively. Data were compared using Fisher’s exact test (B and C) and Student’s t test for (D and E). n represents the number of cells. *p<0.05.

**Fig 6:**
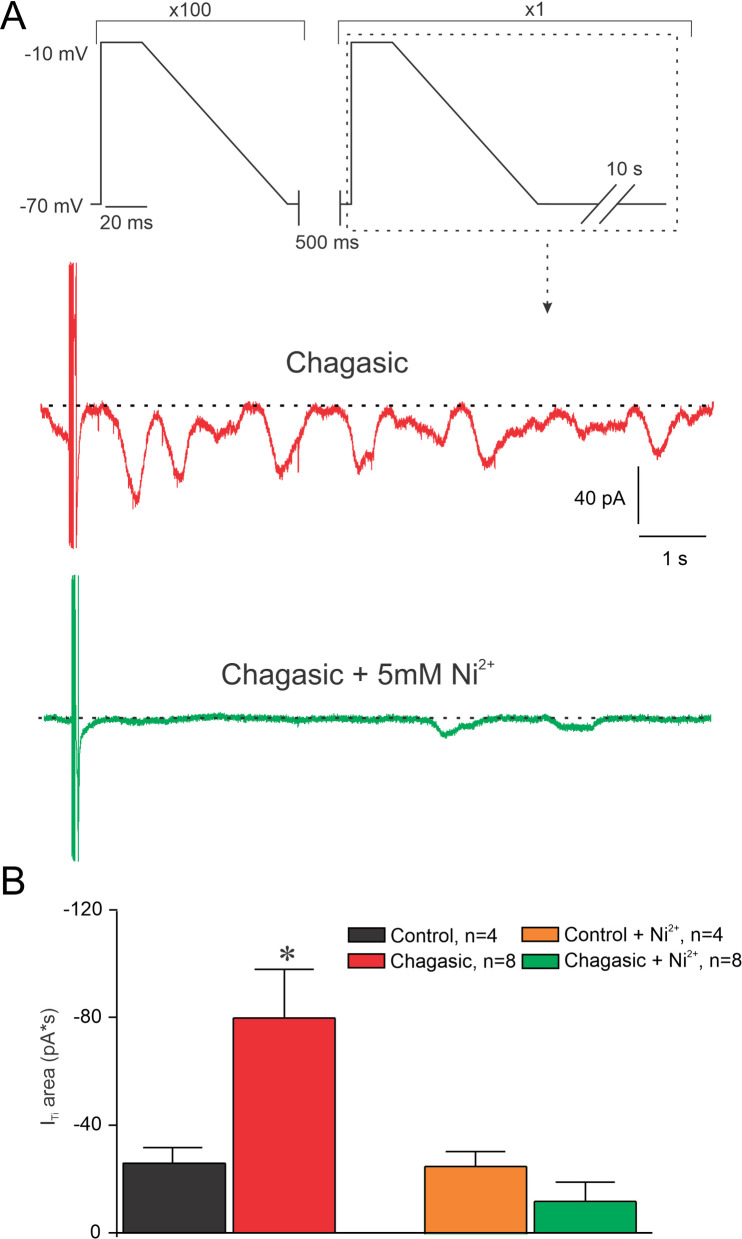
Ni^2+^ attenuates transient inward current (I_Ti_) in the chronic phase of experimental Chagas disease. The protocol is depicted at the top of the figure. (A) Current traces following 100 pre-conditioning pulses and a single 500 ms pause using long pulse, before (red) and after perfusion of 5 mM Ni^2+^ (green traces). (B) Composite data representing the calculated area during I_Ti_ responses recorded from isolated cardiomyocytes from control and infected mice using long AP-simulating pulses, before and after Ni^2+^ perfusion. Data were compared using OneWay ANOVA. n represents the number of cells. *p<0.05.

**Fig 7:**
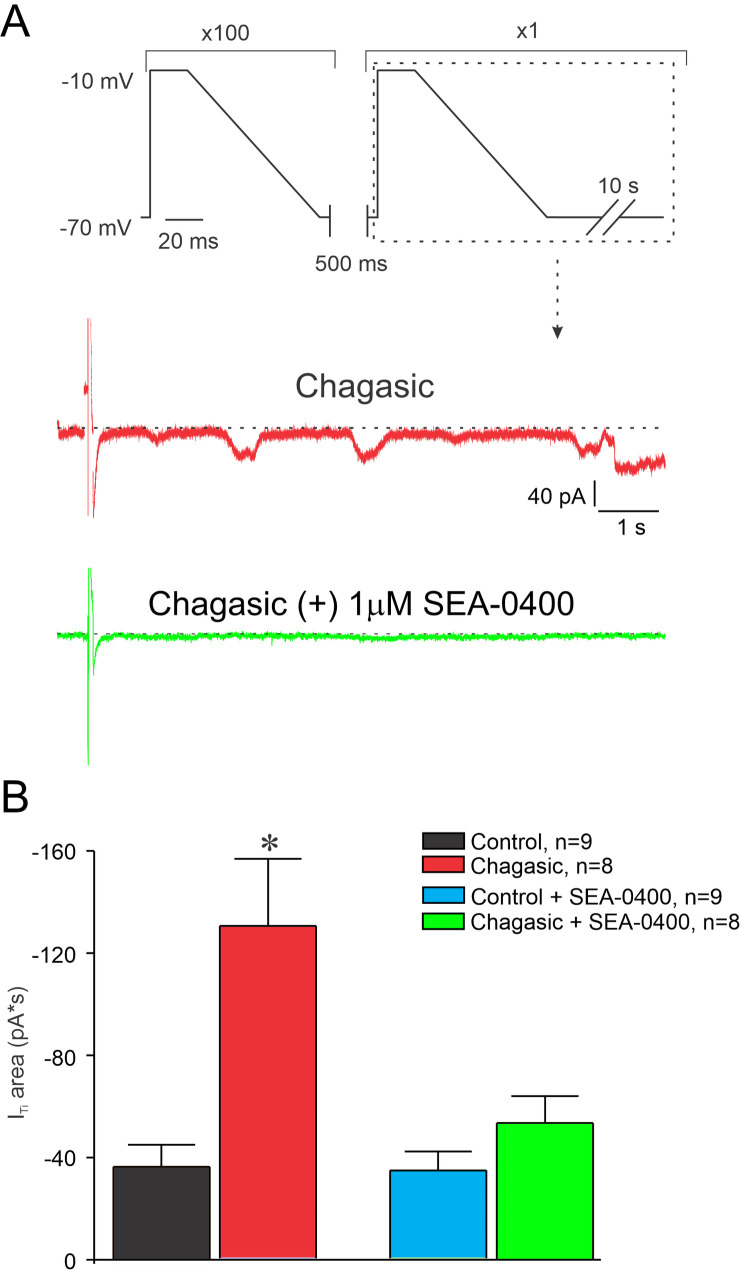
SEA0400 attenuates transient inward current (I_Ti_) in the chronic phase of experimental Chagas disease. The protocol is depicted at the top of the figure. (A) Current traces following 100 pre-conditioning pulses and a single 500 ms pause using long pulse, before (red) and after perfusion of SEA0400 (1 μM). (B) Composite data representing the calculated integral of the I_Ti_ responses recorded from isolated cardiomyocytes from control and infected mice using long pulses, before and after SEA0400 (1 μM). Data were compared using OneWay ANOVA. n represents the number of cells. *p<0.05.

There is evidence in the literature that enhanced I_Ti_ as a result of a stimulation regime of tachycardia following a pause is due to increased Ca^2+^ accumulation into the SR [19]. Thus, we decided to investigate whether I_Ti_ is increased in our experimental model of CD. Fig 5 summarizes our findings. When control and chagasic LVC were stimulated with either short or prolonged AP-like stimulation protocols, both chagasic and control cell groups displayed I_Ti_ but with distinct behavior (Fig 5A). It is important to note that a larger number of infected LVC presented I_Ti_ when compared to controls (Fig 5B and 5C). Also, the total I_Ti_ calculated was larger in infected when compared to control LVC (Fig 5D and 5E). In order to determine whether I_Ti_ is sensitive to Ni^2+^ and SEA0400 we ran the same protocol in another set of cells. First, we applied the protocol depicted on the top of Fig 6A which mimics the prolonged AP waveform typical from chagasic LVC, prior and after extracellular perfusion with 5 mM Ni^2+^. As demonstrated in Fig 6B, the net I_Ti_ is substantially larger in chagasic LVC and it was attenuated to control level after 5 mM Ni^2+^ perfusion, meanwhile I_Ti_ in the control group is insensitive to 5mM of Ni^2+^. A similar protocol was used for 1 μM SEA0400 (Fig 7A) and a similar result was observed for SEA0400 (Fig 7B). Thus, with these experiments, we can conclude that chagasic LVC are more susceptible to generate I_Ti_ sensitive to Ni^2+^ and SEA0400.

### Ion currents in experimental chronic Chagas disease

**Fig 8:**
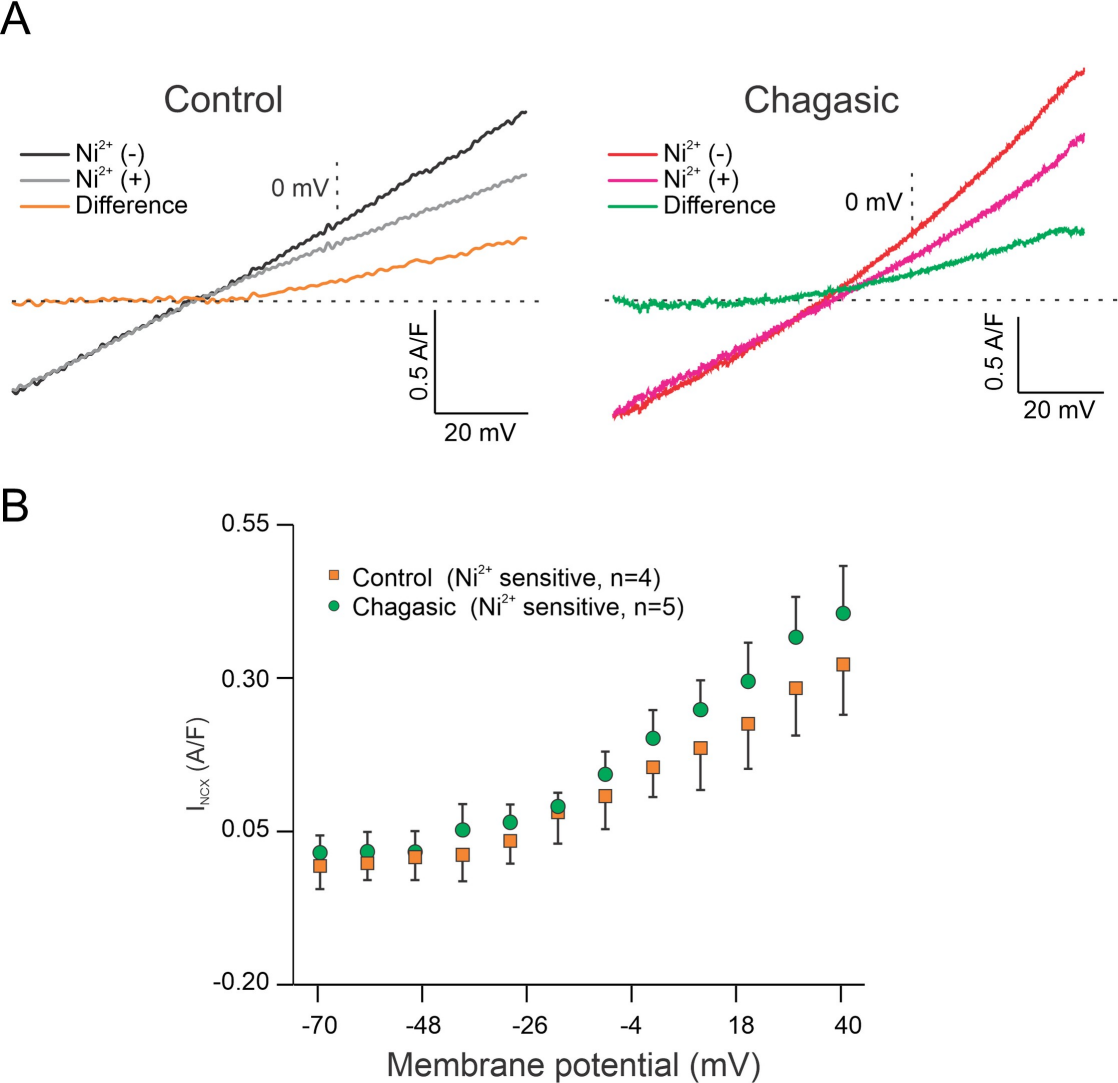
Ni^2+^-sensitive Na^+^/Ca^2+^ exchange current (I_NCX_) density does not change in experimental Chagas disease. (A) Representative I_NCX_ vs. Membrane Potential relationships before (Ni (-)) and after (Ni (+)) perfusion of 5 mM Ni^2+^ measured in isolated cardiomyocytes from control (Panel A, left) and infected (Panel A, right) mice. Orange and green traces represent the difference current obtained by digitally subtracting traces before and after Ni^2+^ application. (B) Average Ni^2+^-subtracted Current Density *versus* Membrane Potential in control (orange squares) and chagasic (green circles) cardiomyocytes were not different. Data were analyzed using Two-way ANOVA. n indicates the number of cells.

**Fig 9:**
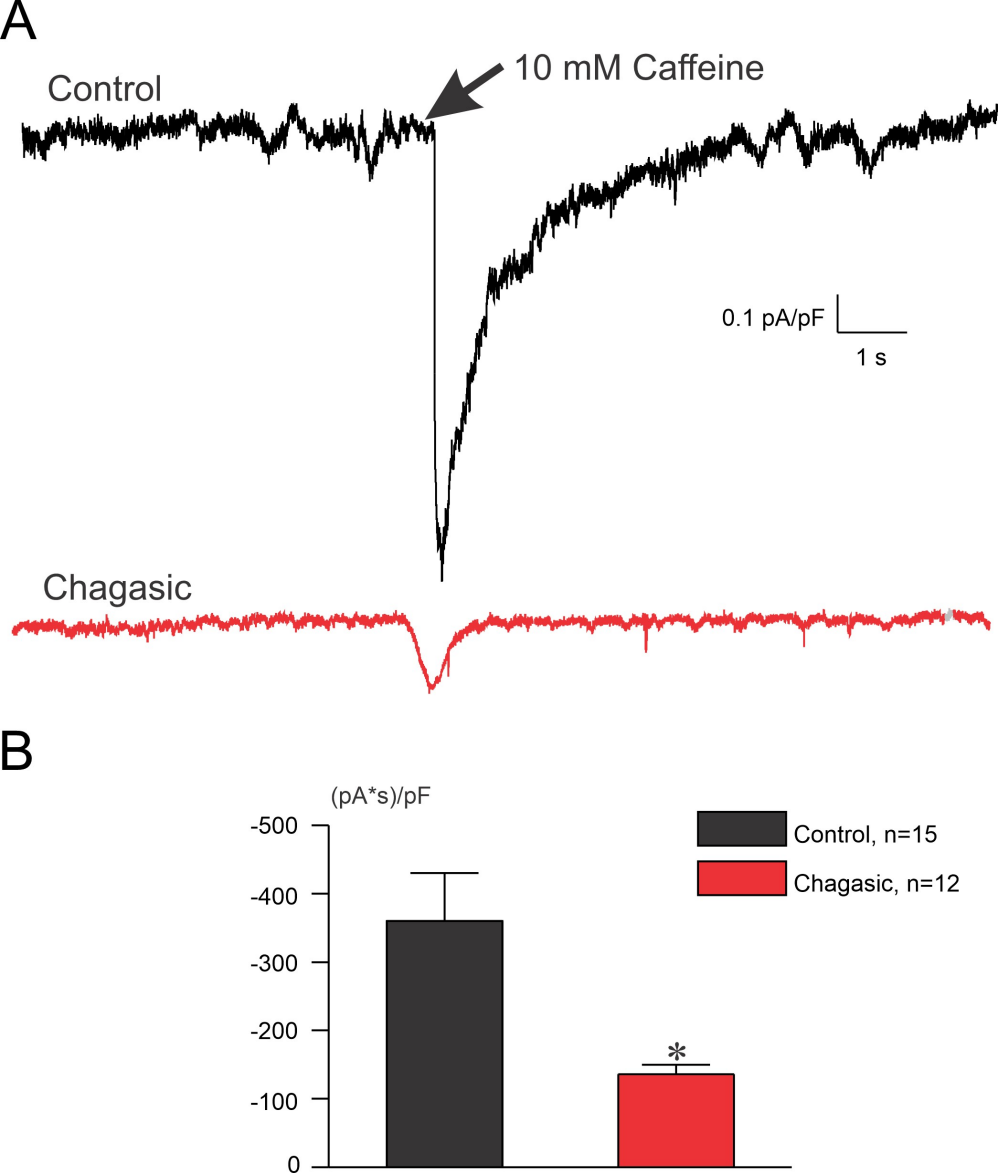
Reduced sarcoplasmic reticulum Ca^2+^ content measured by Na^+^/Ca^2+^ exchange current in the chronic phase of experimental Chagas disease. (A) Representative recordings of caffeine-induced Na^+^/Ca^2+^ exchange current (I_NCX_) in cardiomyocytes from control (black) and infected (red) mice. (B) Area of inward current measured in the presence of 10 mM caffeine. Data were compared using Student’s t test. n indicates the number of cells. *p<0.05.

**Fig 10:**
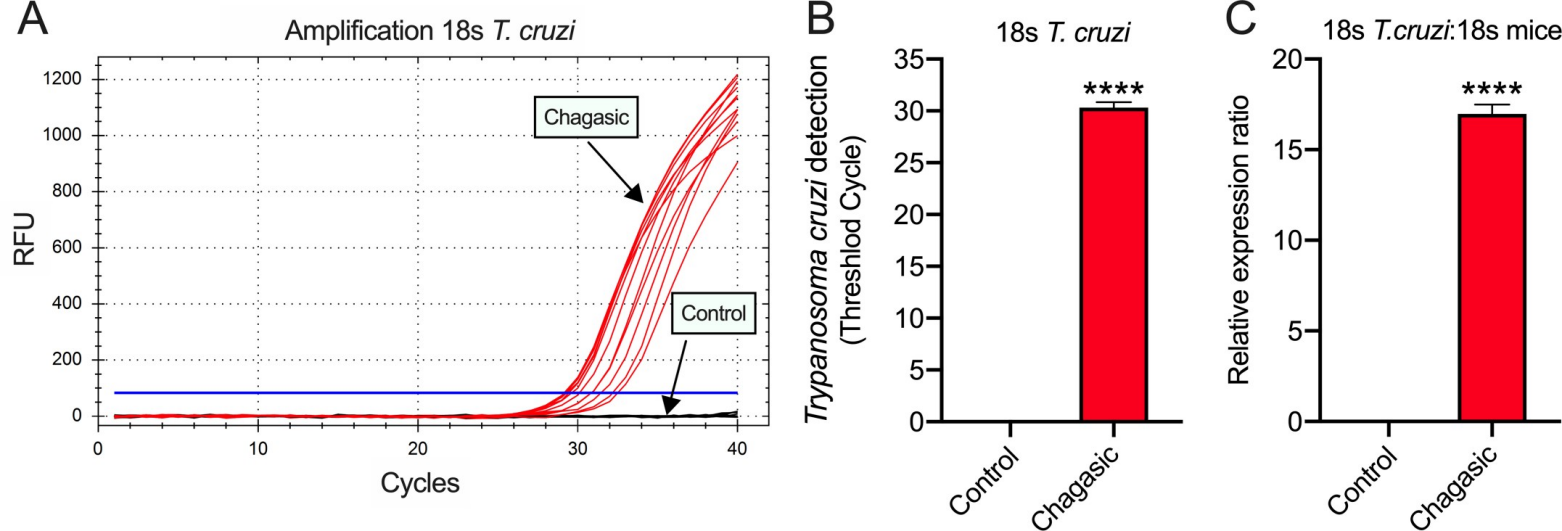
Detection of *T*. *cruzi* in isolated left ventricular cardiomyocytes from mice. RT-PCR analysis was performed using isolated cells obtained from mice 180 to 200 days after infection and age matched controls. The results of RT-PCR amplification of 18S *T*. *cruzi* (A), threshold cycle (B), and relative expression (C; represented as ΔCt values) were demonstrated. Data were normalized to mice 18S endogenous (isolated left ventricular cardiomyocytes). N = 5 for both groups. Data were compared using Student’s t test. ****p<0.0001.

In the literature it is well documented that I_NCX_ is sensitive to Ni^2+^ [17]. During the ramp protocol, the membrane potential was initially held at −30 mV to inactivate Na^+^ channels. Cells were then depolarized to +40 mV to induce an outward current (reverse mode of NCX), as seen in Fig 8. The current becomes inward (forward mode of NCX) as the cell is hyperpolarized to −70 mV. The protocol was repeated in the presence of Ni^2+^ to yield what we defined as Ni^2+^-sensitive current which was interpreted as I_NCX_. Fig 8A shows representative tracings of I_NCX_ from control (left traces) and chagasic (right traces) LVC. The mean population data of peak outward current density (at +40 mV) reveals that I_NCX_ was similar in control and chagasic LVC (p>0.05). Also, as indicated by our analysis from -70 to +40 mV (Fig 8B) the net I_NCX_ is similar in control and chagasic LVC. In order to further explore this issue, we assessed Na^+^/Ca^2+^ exchanger activity in patch-clamped LVC by rapidly applying 10 mM caffeine to the bath solution while recording membrane current at a holding potential set at -80 mV. To ensure a steady SR loading, cells were pre-pulsed as indicated in the method section. In control LVC, the application of caffeine induced a large inward current, as depicted in Fig 9A (Top trace). However, in chagasic LVC, caffeine-induced inward current was significantly smaller (Fig 9A, Bottom trace). Fig 9B shows composite data. These functional data are consistent with reduced SR Ca^2+^ content as suggested in previous study [10]. Finally, using RT-PCR analysis and endogenous 18S mice for internal normalization, the detection of *T*. *cruzi* in isolated LVC from infected mice was demonstrated. The results of RT-PCR amplification of 18S *T*. *cruzi* are shown in Fig 10A, the threshold cycle (Fig 10B), and relative expression (Fig 10C).

## Discussion

Cardiac arrhythmia is a common trait in the chronic phase of CD and it is also a common finding in experimental murine models of CD [5,6,9]. Arrhythmias are determined by examination of the surface electrocardiogram that reveals, for instance, premature ventricular beats, sustained and non-sustained ventricular tachycardia, which may be attributed to altered cardiomyocyte excitability [19,20]. In the last decade, a substantial advance in the understanding of the cellular basis for ventricular arrhythmias in CD occurred, taking advantage of experimental animal models. However, major gaps in the field remain to be addressed. In the present investigation, we found a new cellular arrhythmogenic substrate in a murine model of CD, which can enable us to pursue new and more specific therapeutic approaches in future studies.

In previous studies using a murine model of CD, it was found profound alteration in cellular excitability of LVC during the time-course of the disease [6], and this was associated with the production of inflammatory cytokines [6,12]. Importantly it was described an AP prolongation that was linked to the reduction of voltage-dependent K^+^ currents [6,7]. In these previous studies, however, the use of Ca^2+^ chelator into the patch-clamp pipette during AP recordings probably diminished the surge of Ca^2+^ -dependent conductances in the AP waveform. This maneuver likely prevented the appearance of pro-arrhythmogenic events, such as after-depolarizations and could further underestimate the remodeling of AP waveform after *T*. *cruzi* infection. Thus, we decided to revisit the AP waveform, but now measuring it without adding a Ca^2+^ chelator into the patch-clamp pipette. Under these conditions, we did observe the appearance of afterdepolarizations that culminate in spontaneous AP, which could be triggered by I_Ti_ [19,21]. Also, I_Ti_ has long been recognized to be arrhythmogenic, underlying transient membrane depolarizations in conditions of intracellular Ca^2+^ overload [19,22]. I_Ti_ is dependent on intracellular Ca^2+^ concentration [23,24], a condition that we have previously shown to be increased in cardiomyocytes isolated from chagasic mice in the acute phase (30–45 days post-infection [9]). In line with this idea, diastolic Ca^2+^ overload is also a hallmark of cardiomyocytes from humans with CCM [25].

In order to better explore the role of APR prolongation in arrhythmogenesis in LVC we simulated two situations for both groups of cells: a train of AP-like voltage-clamp with (1) control and (2) chagasic features. By using the first simulated condition we observed that a greater number of chagasic LVC developed I_Ti_ showing larger amplitude when compared to controls. For the second simulated condition, we continued to observe a greater number of chagasic LVC developing I_Ti_ when compared to controls. Combined these results indicate that only rescuing AP waveform may be not enough to prevent arrhythmogenesis in CD.

On the other hand, reduction of AP duration and membrane potential instabilities were observed when chagasic LVC were exposed to Ni^2+^. Thus, Ni^2+^ likely does both reduce AP prolongation and attenuate I_Ti_. It is well known that Ni^2+^ is used to study Na^+^/Ca^2+^ exchanger function in cardiomyocytes [26,27]. However, it is relevant mentioning that Ni^2+^ can have off-target effects on ionic conductances other than the blockage of Na^+^/Ca^2+^ exchanger with variable selectivity, including the blockage of Na^+^ current [28], and of several types of Ca^2+^ current, with higher affinity for T-type compared to L-type Ca^2+^ channels [29–31]. Ca^2+^ dynamics has an important participation in shaping AP waveform and can be an important determinant of arrhythmogenic profile such as AP alternans [32]. Hence, the off-target effects of Ni^2+^ could be overestimating the contribution of Na^+^/Ca^2+^ exchanger on remodeled AP from chagasic mice.

Nevertheless, our findings were further supported using a selective blocker of I_NCX_, SEA0400. To our surprise, I_NCX_ density was comparable in both studied groups. It is worth to mention that in I_NCX_ recordings, [Ca^2+^]_i_ was maintained at 152 nM, which excluded the modulatory effect of Na^+^/Ca^2+^ exchanger by [Ca^2+^]_i_ [33]. However, an increase in LVC diastolic [Ca^2+^]_i_ as previously described by our group in chagasic mice [9], and in humans with CD [25], may lead to I_NCX_ activation [34], accounting for the observed prolongation of AP duration at more negative membrane potentials. It is important to note that using both blockers, Ni^2+^ and SEA0400, the fractional shortening of APR duration at T90 was similar in both, control and chagasic LVC. The result may suggest that prolongation of AP duration, as a consequence of reduced transient inward potassium current, as already reported in previous studies [7,8,10], favors enhanced contribution of I_NCX_ without increasing its current density. Further experiments are necessary to clarify this question.

Our results also point towards a reduction of SR Ca^2+^ load, as indicated by the reduction of caffeine-induced Ca^2+^ release from SR. This is in accordance with previous findings of reduced SERCA2A activity extrapolated from global Ca^2+^ transient decay time [6,10]. In this study, we strengthened the idea that Ca^2+^ dynamics dysfunction has an important role in the electrical remodeling of cardiomyocytes during experimental CD, as it was previously implicated in cardiomyocytes isolated from patients with CD [25]. The tachycardia-like protocol favors Ca^2+^ release from SR and, along with reduced SERCA2A function, would contribute to Ca^2+^ accumulation in the sarcoplasm. In the end, a prolonged AP in chagasic LVC elicited an increase in I_NCX_ at the diastolic level, explaining the fact that Ni^2+^ and SEA0400 reduced the pause-induced diastolic I_Ti_. Lastly, since the molecular identification of *T*. *cruzi* in isolated LVC from chagasic mice was found, we may speculate that parasite persistence in the heart is an important component to the development of CCM, which would favor a chronic inflammation in the tissue, and then contribute to the observed electrical remodeling observed [35–37]. Future experiments are needed to access the putative association between parasite persistence and electrical remodeling in experimental CCM.

## Conclusion

In the present study, we demonstrate that in the chronic phase of experimental infection with *Trypanosoma cruzi* (TcI, Colombian strain), LVCs have increased AP duration and they are more susceptible to display early and delayed after-depolarizations, leading to increased frequency of cells showing spontaneous AP. Moreover, diseased LVCs are more prone to present pause-induced I_Ti_. Most importantly, the arrhythmogenic mechanisms underlying these events are sensitive to Ni^2+^ and SEA0400, which certainly indicates the involvement of I_NCX_. Thus, inhibiting I_NCX_ could be a potential and promising therapeutic strategy for the prevention of ventricular arrhythmias found in Chagasic cardiomyopathy.

### Study limitations

First, we conducted our cellular electrophysiological experiment at room temperature. Thus, the extrapolation of Ca^2+^ dynamics influence *in vivo* is limited. Second, we speculate that off-target effects of Ni^2+^ other than a block of I_NCX_ may also contribute to the antiarrhythmic effect. Also, we did not access the fraction of cardiomyocytes infected or not with *T*. *cruzi*, since cellular electrical remodeling was investigated as a result of the net heart impairment after chronic infection with *T*. *cruzi*. Further studies are necessary to resolve these issues.

## Supporting information

S1 Table Solutions used in patch-clamp experiments. (DOCX)

S2 Table Action potential parameters. (DOCX)

S1 Data Underlying data for the reported findings. (XLSX)
