# Supplementary material for: A novel substrate for arrhythmias in Chagas disease
Source: PLoS Negl Trop Dis. 2021 Jun 2;15(6):e0009421. doi: 10.1371/journal.pntd.0009421 (PMC8172059; doi:10.1371/journal.pntd.0009421)
Supplement: S1 Table — (DOCX) [file pntd.0009421.s001.docx]

**Supporting Information**

**S1 Table:** Solutions used in patch-clamp experiments.

| Supplementary Table 1 – Solutions used in patch-clamp experiments | 1^#^ | 2^#^ | 3^#^ | 4^#^ |
| --- | --- | --- | --- | --- |
| Aspartic Acid |  | 130 |  |  |
| BaCl_2_ |  |  |  | 1 |
| CaCl_2_ | 1.8 |  | 29 | 2 |
| CsCl |  |  | 20 | 2 |
| CsOH |  |  | 160 |  |
| EGTA |  |  | 42 |  |
| Glucose | 11 |  |  | 5.5 |
| HEPES | 5 | 10 |  | 10 |
| Mg-ATP |  |  | 10 |  |
| MgCl_2_ | 0.5 | 2 |  | 2 |
| Nifedipine |  |  |  | 0.01 |
| NiCl_2_ |  |  |  | 5 |
| NaH_2_PO_4_ | 0.33 |  |  | 0.33 |
| Ouabain |  |  |  | 0.02 |
| KCl | 5.4 | 20 |  |  |
| KOH |  | 130 |  |  |
| NaCl | 140 | 5 |  | 140 |
| NaOH |  |  | 20 |  |
| pH | 7.4 w/ NaOH | 7.2 w/  KOH | 7.2 w/ CsOH | 7.4 w/ NaOH |

^#^All concentrations are given in mM. 1 - Tyrode; 2- internal solution for action potential and transient inward current; 3 – internal solution for Na^+^/Ca^2+^ exchanger current; 4 – external solution for Na^+^/Ca^2+^ exchanger;
