# Supplementary material for: A novel substrate for arrhythmias in Chagas disease
Source: PLoS Negl Trop Dis. 2021 Jun 2;15(6):e0009421. doi: 10.1371/journal.pntd.0009421 (PMC8172059; doi:10.1371/journal.pntd.0009421)
Supplement: S2 Table — (DOCX) [file pntd.0009421.s002.docx]

**Supporting Information**

**S2 Table:** Action potential parameters

|  | $\frac{dV}{dt}$ (V/s) | Overshoot (mV) | Resting membrane Potential (mV) |
| --- | --- | --- | --- |
| Control 0.2 Hz | 170.6 ± 16.7 | 37.7 ± 2.4 | -67.6 ± 0.7 |
| Chagasic 0.2 Hz | 166.7 ± 8.5 | 38.7 ± 1.6 | -65.5 ± 0.3* |
| Control 1.0 Hz | 163.3 ± 27.8 | 38.5 ± 2.7 | -65.4 ± 0.5 |
| Chagasic 1.0 Hz | 182.1 ±11.2 | 45.7 ± 1.9 | -64.2 ± 0.7 |

*p < 0.05, Chagasic vs. Control.
